# The Effects of Age and Cell Isolation on Collagen II Synthesis by Articular Chondrocytes: Evidence for Transcriptional and Posttranscriptional Regulation

**DOI:** 10.1155/2020/4060135

**Published:** 2020-04-30

**Authors:** Vipin Asopa, Tonia Vincent, Jeremy Saklatvala

**Affiliations:** Centre for OA Pathogenesis Versus Arthritis, Kennedy Institute of Rheumatology, University of Oxford, Roosevelt Drive, Headington, Oxford OX3 7FY, UK

## Abstract

Adult articular cartilage synthesises very little type II collagen in comparison to young cartilage. The age-related difference in collagen II synthesis is poorly understood. This is the first systematic investigation of age-related differences in extracellular matrix synthesis in fresh articular cartilage and following isolation of chondrocytes. A histological comparison of 3-year-old skeletally mature and 6-month-old juvenile porcine cartilage was made. Differences in collagen II, aggrecan, and Sox5, 6, and 9 mRNA and protein expression and mRNA stability were measured. Adult cartilage was found to be thinner than juvenile cartilage but with similar chondrocyte density. Procollagen *α*1(II) and Sox9 mRNA levels were 10-fold and 3-fold reduced in adult cartilage. Sox9 protein was halved and collagen II protein synthesis was almost undetectable and calculated to be at least 30-fold reduced. Aggrecan expression did not differ. Isolation of chondrocytes caused a drop in procollagen *α*1(II) and Sox9 mRNA in both adult and juvenile cells along with a marked reduction in Sox9 mRNA stability. Interestingly, juvenile chondrocytes continued to synthesise collagen II protein with mRNA levels similar to those seen in adult articular cartilage. Age-related differences in collagen II protein synthesis are due to both transcriptional and posttranscription regulation. A better understanding of these regulatory mechanisms would be an important step in improving current cartilage regeneration techniques.

## 1. Introduction

In 1744, William Hunter wrote of articular cartilage, that “when destroyed, it is never recovered” [1]. While this is true of the tissue in adults, fetal and juvenile cartilage has the potential to heal. Surgically created defects in fetal articular cartilage can spontaneously heal [2, 3], and fetal articular cartilage homografts transplanted into adult rabbit femoral condyles can integrate completely into the surrounding articular cartilage [4]. Cartilage regeneration has been reported following articular cartilage injury in 3-month-old rabbits, but not in adult animals [5]. This inability of adult cartilage to regenerate could be due to either or both reduced proliferative or extracellular matrix synthetic capacities of adult chondrocytes [6]. Hermansson et al. [7] reported that cultured explants of human osteoarthritic or infant articular cartilage synthesised significant amounts of type II collagen protein, whereas healthy adult cartilage synthesised very little or none. Monolayer cultures of articular chondrocytes isolated from human specimens of subjects aged over 40 show reduced proliferation compared to those from younger individuals [8]. Furthermore, culture and passaging in monolayer results in dedifferentiation of chondrocytes to fibroblast-like cells with loss of collagen II protein and proteoglycan expression [9]. Absence of growth factors does not account for these changes, but they are partially reversible in 3-dimensional culture [10]. This tendency of chondrocytes to lose their phenotype may explain the limited benefit of current cell-based therapies for cartilage injuries.

From a histological perspective, studies such as those by Stockwell and Scott reported reduced territorial staining of proteoglycan in older human costal cartilage [11] and Francuski et al. [12] recently reported thinning of articular cartilage in adult German Shepherd dogs, although immunohistochemical staining for collagen II protein was the same in adult and juvenile dogs. To date, there has been very little published on the age-related differences in the synthesis of cartilage-specific molecules in articular cartilage, nor has the effect of isolation of adult and juvenile chondrocytes upon their ability to make extracellular matrix been compared.

The purpose of the work described in this paper was firstly to measure the differences in mRNA and protein expression of type II collagen and aggrecan, the main proteoglycan, and the regulatory transcription factors Sox5, 6, and 9 between adult and juvenile articular cartilage. Secondly, our work investigates the change in expression of type II collagen caused by removing chondrocytes from their normal milieu.

Due to the difficulty of obtaining human articular cartilage samples of the same age and site of origin, we were forced to use animal tissue. We chose to use pigs for the study because their joints are of a similar size to human, and their forefeet, which are easily obtained from an abattoir, provide cartilage from weight-bearing joints of animals of specific ages, thus allowing replication of samples.

## 2. Methods

### 2.1. Tissue

Forefeet (trotters) of freshly slaughtered pigs were obtained from an abattoir. Articular cartilage was dissected from metacarpophalangeal joints of male and female 3-month old pigs and 3-year old adult breeding sows.

### 2.2. Reagents

Antibodies used were to extracellular regulated kinase (ERK) (no. 9122; New England Biolabs, Beverly, MA), Sox5 (AB26041), Sox6 (AB12054; Abcam, UK), and Sox9 (AB5535; Chemicon International, USA). Mouse hybridoma monoclonal anticollagen II (CN X) antibody (AET) was a gift from Professor Vic Duance, University of Cardiff, UK, and has been validated for use in pigs [13]. Secondary antibodies were purchased from Dako (Glostrup, Denmark).

Enhanced chemiluminescence (ECL) reagents and [^35^S] methionine (Met)/[^35^S] cysteine (Cys) were from Amersham Biosciences; pronase E was from BDH Chemicals, Poole, UK; DMEM and fetal calf serum (FCS) were from Biowhittaker, Berkshire, UK; and horseradish peroxidase-conjugated (HRP) and rabbit anti-mouse IgG were from Dako A/S, Glostrup, Denmark. Other reagents, unless specified, were from Sigma-Aldrich Company Ltd., Dorset, UK.

### 2.3. Histological Analysis

Sections of articular cartilage and underlying bone were made from identical matched locations of adult and juvenile distal metacarpophalangeal joints, which had been fixed in formalin, decalcified, and embedded in paraffin wax. Sections from 6 adult and 6 juvenile animals were stained with haematoxylin and eosin (H&E) and with Safranin O at the same time.

### 2.4. Tissue Culture

Cartilage explants (50 mm^3^) were dissected into culture medium (DMEM with 25 mM HEPES (N-(2-hydroxyethyl)-piperazine-N′-2-ethanesulfonic acid) buffer (pH 7.4), penicillin (100 units/ml), streptomycin (100 *μ*g/ml), amphotericin (2 *μ*g/ml), and 200 *μ*M ascorbic acid 2-phosphate, 2 ml/g) of tissue. Chondrocytes were cultured in the same medium with 10% FCS.

### 2.5. Isolation of Chondrocytes

Chondrocytes were isolated using pronase E (1 mg/ml/g of cartilage) for 30 min, followed by collagenase (1 mg/ml/g) for 5 h at 37°C. The digest was strained (70 *μ*m) and centrifuged (500 x g, 8 min). Pellets were washed twice and resuspended in culture medium. Cells were plated on 24-well plates (1.5 cm diameter) at a density of 1 million cells per well. Isolated chondrocytes were cultured with 10% FCS and incubated in a humidified atmosphere of 95% air and 5% CO_2_ at 37°C. Confluence was 90-100%.

### 2.6. Metabolic Labelling of Newly Synthesised Proteins

Chondrocytes were rested for 18 h, and then washed in Met/Cys-free medium for 1 h to starve the cells of the two amino acids in order to facilitate their take up when incubated with 15 *μ*Ci/well of [^35^S] Met/[^35^S] Cys in fresh medium. After 6 h of incubation, the medium was removed, centrifuged (10,000 x g, 5 min), mixed with sample buffer, electrophoresed (12.5% SDS-PAGE), and silver stained. Newly synthesised proteins were visualised by autoradiography and silver-stained bands corresponding to radiolabelled ones excised and identified by mass spectrometry [7].

### 2.7. Western Blot for Intracellular Sox5, Sox6, Sox9, and ERK in Isolated Chondrocytes and Cartilage Explants

For chondrocytes, each well was washed with ice-cold phosphate-buffered saline, then lysed with 250 *μ*l of ice-cold radioimmunoprecipitation assay (RIPA) buffer following a standard protocol (20 mM Tris-HCl (pH 7.4), 150 mM NaCl, 5 mM EDTA, 1% (volume/volume) Triton X-100, 0.1% (weight/volume) SDS, 1% (*w*/*v*) sodium deoxycholate, 0.5% (*v*/*v*) Igepal CA-630, 1 mM phenylmethylsulfonyl fluoride, 10 *μ*M E64, 1 *μ*g/ml pepstatin A, and 10 *μ*g/ml aprotinin) [13]. Lysates were removed after 45 min and centrifuged (14,000 x g, 15 min). Protein concentration was determined by the Bradford colorimetric procedure (Bio-Rad) [14]. 15 *μ*g of protein was mixed with 4x sample buffer, boiled (5 min) and electrophoresed (precast 4-12% bis-tris gradient, Invitrogen NuPAGE (Thermo Fisher Scientific), UK), transferred to polyvinylidene difluoride (PVDF) membrane (Millipore, Bedford, MA) and blocked (30 min) in 5% dried milk. Membranes were treated with either rabbit-polyclonal anti-human Sox5, Sox6, or Sox9 primary antibody for 1 h, washed 3 times (PBS+0.05% Tween 20), and then incubated for 1 h with HRP antibody. Antibody staining was visualised with enhanced chemiluminescence (ECL). Expression of multiple proteins, including the loading control ERK, was achieved by stripping antibodies with 100 mM 2-mercaptoethanol, 2% SDS, and 62.5 mM Tris-HCl (pH 6.7) for 30 min at 50°C with stirring, and reprobing.

For cartilage explants, 1 g of cartilage tissue was dissected into 2 ml of ice-cold 15 mM HEPES buffer and snap frozen in liquid nitrogen for 5 min and thawed at 37°C 3 times. Cellular protein was extracted using RIPA at 4°C for 2 h with agitation. Extracts were centrifuged (13,000 x g, 20 min). Protein concentration was determined, and samples were prepared and analysed as above.

### 2.8. Western Blot for Extracellular Collagen II Secreted into Culture Medium

Isolated chondrocytes were cultured for 18 h then washed in serum-free medium and cultured for a further 6 h. Cartilage explants were cultured in serum-free medium for 18 h, washed, and then cultured for a further 6 h. This medium was then harvested and protein concentration was determined. Aliquots of the culture medium and samples were then subjected to SDS-PAGE (fresh tris-glycine 8%, 12% gels cast in 13 cm glass plates), electrotransferred to PVDF, and reacted with anticollagen II antibody as previously described [15]. Visualisation was with a secondary antibody and ECL.

### 2.9. Quantification of Protein Expression

Autoradiographs were scanned using a Bio-Rad 710 imaging densitometer and analysed using Phoretix 2D software (version 6.01; Nonlinear Dynamics Ltd., Newcastle, UK). The area and pixel intensity of each band were measured, and individual spot volumes were calculated.

### 2.10. Real Time Reverse Transcriptase Polymerase Chain Reaction on Cartilage Explants and Isolated Chondrocytes

One gram of cartilage was homogenized with a POLYTRON Rotor-Stator homogenizer (Kinematica, Luzern, Switzerland) in 2 ml of TRI Reagent (Helena Biosciences, Gateshead, UK), and RNA was extracted using chloroform. The aqueous phase was mixed with an equal volume of 70% (*v*/*v*) ethanol and processed using the QIAamp RNA Blood Mini Kit (Qiagen, Chatsworth, CA). One microgram of Total RNA was reverse transcribed using SuperScript II (Invitrogen, San Diego, CA). For chondrocytes, RNA was isolated using the QIAamp RNA Blood Mini Kit with a DNAse step.

cDNA was amplified with specific primers for GAPDH (forward 5′-GATCGAGTTGGGGCTGTGACT-3′, reverse 5′-ACATGGCCTCCAAGGAGTAAGA-3′), *β*-actin (forward-5′-CTCGATCATGAAGTGCGACGT-3′, reverse-5′-GTGATCTCCTTCTGCATCCTGTC-3′), and aggrecan (forward-5′-CCAGAATCTAGCAGGGAGTCATC-3′, reverse-5′-AGGCAGAGGTGGCTTCAGTC-3′) in standard polymerase chain reactions (PCR) containing SYBR-green master mix (MWG Biotechnology, UK). For samples of 10 *μ*l, conditions used were initial denaturation at 50°C for 2 min, 95°C for enzyme activation for 10 min, then 40 cycles of denaturation at 95°C for 15 sec and annealing and extension at 60°C for 1 min.

For procollagen *α*1(II) (probe 6-FAM-ACCAGGAACGCCCTGATCACCTGG-TAMRA, forward-5′-CCATCTGGCTTCCAGGGAC-3′, reverse-5′-CCACGAGGCCAGGAGCT-3′), Sox9 (Hs00165814_m1, probe 5′-GAGCACTCGGGGCAATCCCAGGGC-3′), Sox6 (Hs00264525_m, probe 5′-AACAGCAAGAACAGATTGCGAGACA-3′), Sox5 (Hs00374709_m1, probe 5′-ACGAGCCGGAAGAAACCCCCAGTAT-3′), and 18S (Hs99999901_s1, probe 5′-GGAGGGCAAGTCTGGTGCCAGCAGC-3′), a sample volume of 25 *μ*l was used in TaqMan® Master Mix (Applied Biosystems (Thermo Fisher Scientific), UK). Conditions were initial denaturation at 50°C for 2 min, then 40 cycles of denaturation at 95°C for 15 sec, annealing, and extension at 60°C for 1 min.

Real time PCR was performed using a Rotor-Gene 6500 (Corbett Research, Australia). Specificity and efficiency were confirmed by serial dilutions, melting curves, and visualisation of PCR products on an agarose gel. Data was averaged from triplicates and results shown graphically with bars representing standard error of the mean (SEM). The SEM or variation of reference was calculated using $\sqrt{\left[\text{SEM }{\left(\text{control}\right)}^{2}+\text{SEM }{\left(\text{test sample}\right)}^{2}\right]}$. Statistical analysis was performed using Student's *t*-test (Prism software v4.03), and significance was taken as at least 2-fold difference, with SEM error bars not crossing base line.

The suitability of housekeeping genes GAPDH, *β*-actin, and 18S was confirmed by constant amounts measured in 1 *μ*g RNA in adult and juvenile, male and female pigs. Results with Tacman PCR reagents were normalized to 18S and with conventional, to *β*-actin.

### 2.11. Determination of mRNA Stability

Isolated chondrocytes were cultured for 48 h and transcription inhibited using actinomycin D (10 *μ*g/ml). Fresh cartilage explants were dissected into DMEM containing actinomycin D (10 *μ*g/ml). mRNA levels were determined as described above.

## 3. Results

Domestic pigs reach sexual maturity at 6 months of age, reach skeletal maturity at 18 months, and have a life expectancy of up to 25 years [16, 17]. Juvenile tissue was obtained from male and female animals aged 3-6 months, and adult tissue was obtained from 3-year-old breeding sows.

### 3.1. Histology of Adult Cartilage Shows Thinning, Surface Fibrillation, and Reduced Proteoglycan Staining

**Figure 1:**
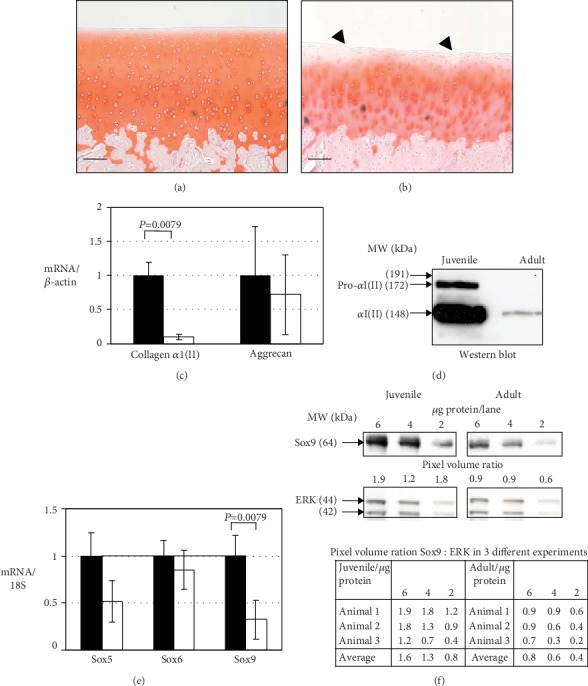
Histological sections from juvenile (a) and adult (b) porcine articular cartilage. Expression of procollagen *α*1(II) and aggrecan mRNA (c), collagen (d) and Sox9 proteins (f), and Sox5, Sox6, and Sox9 mRNA in juvenile (solid bars) and adult (clear bars) (e). (a and b) Sections of articular cartilage were stained with H&E/Safranin O. Sections shown are representative of 6 adult and 6 juvenile animals. Arrowheads show fibrillation in adult cartilage (b). 10x magnification, scale bar = 100 *μ*m. (c and e) For mRNA determination, cDNA templates were prepared from fresh cartilage and real time RT-PCR carried for collagen *α*1(II) and aggrecan (c), and Sox5, Sox6, and Sox9 (e). Relative gene expression levels were analysed using the Mann-Whitney *U* test. Results are means of separate determinations on samples from 6 adult and 6 juvenile animals. (d) To investigate synthesis of collagen II protein from explants, culture medium was replaced with fresh medium after 18 h. The culture was continued for a further 6 hours. Aliquots of the medium (containing secreted protein) were electrophoresed and then western blotted with collagen II antibody. The result shown is representative of 3 separate experiments. Molecular weights indicated are calculated from the migration of marker proteins. (e) Intracellular Sox9 and ERK protein were extracted by freeze-thawing cartilage explants and detected by western blotting. Blots shown are representative of three separate experiments. A ratio of Sox9 : ERK protein intensity was calculated from densitometry for the 3 experiments. Pixel volume ratios are shown in the table.

Vertically cut sections of articular cartilage from adult and juvenile porcine metacarpophalangeal joints were stained with Safranin O. Representative sections are shown in Figure 1. Analysis of 8 sections taken from the same location in each joint show that adult cartilage is 30% thinner: average thickness 425 *μ*m for juvenile cartilage and 303 *μ*m for adult cartilage. The cell density of the juvenile and adult specimens is not significantly different (juvenile cartilage = 1725 ± 460 and adult cartilage = 1830 ± 404 cells per mm^2^). In adult cartilage, there are fewer chondrocytes at the surface, which was uneven and slightly fibrillated (arrowheads, Figure 1(b)). The Safranin O staining, which indicates the content and distribution of proteoglycan (mainly aggrecan), is uniform in the juvenile tissue except for the most superficial region. In the adult tissue, staining is markedly reduced at the surface (5 cells deep vs. 1 cell deep in the juvenile tissue) and in the interterritorial regions of the chondrocytes compared with the pericellular zones. The osteochondral junction is smoother in the adult cartilage compared with juvenile cartilage.

### 3.2. Juvenile Articular Cartilage Synthesises at Least Ten-Fold More Collagen II than Adult

To investigate the difference in collagen II expression between juvenile and adult cartilage, mRNA levels were measured in extracts of freshly dissected tissue. Results of real time RT-PCR for procollagen *α*1(II) mRNA and *β*-actin, which was measured as a housekeeping gene, are shown in Figure 1(c). Levels of procollagen *α*1(II) mRNA were 10-fold greater in juvenile than in adult tissue. To investigate whether the difference in mRNA levels was reflected in synthesis and secretion of collagen II protein, samples of culture medium conditioned for 6 hours from equally sized explants were western blotted with a monoclonal antibody to the protein (Figure 1(d)). The juvenile cartilage medium contained a major band at the migration position expected for the processed procollagen *α*1(II) chain of collagen II. A fainter slower band corresponded to the unprocessed procollagen *α*1(II) chain. The medium from the adult tissue contained only a faint band in the processed procollagen *α*1(II) position. The amount of collagen II secreted was estimated to be between 20- and 30-fold greater in juvenile tissue.

**Figure 2:**
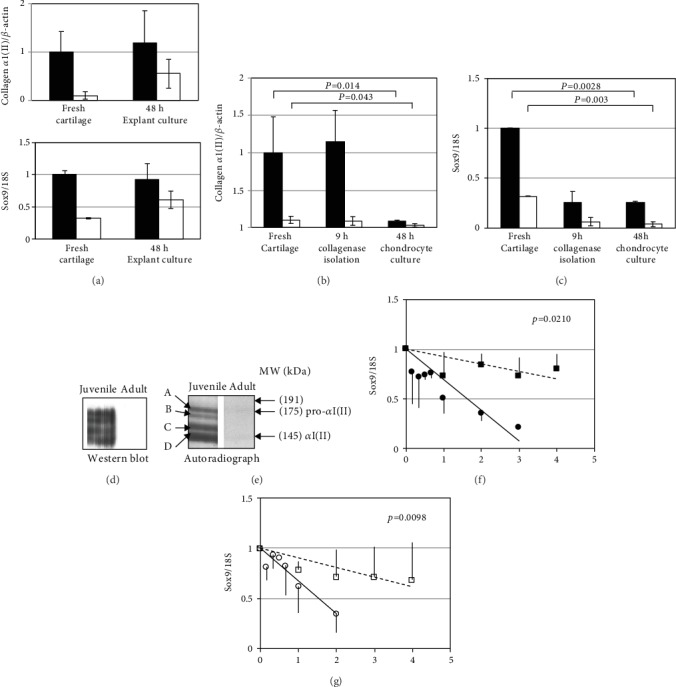
Effect of explantation of adult (clear symbols) and juvenile (solid symbols) cartilage on procollagen *α*1(II) and Sox9 mRNA (a); effect of isolation and culture of adult and juvenile chondrocytes on procollagen *α*1(II) mRNA (b) and Sox9 mRNA (c). Collagen protein synthesis by adult and juvenile chondrocytes (d and e). Sox9 mRNA stability in fresh explants (squares) and isolated chondrocytes (circles) in juvenile (f) and adult (g) chondrocytes. (a, b, and c) mRNA was extracted from either fresh cartilage, explants cultured for 48 h (a), freshly isolated chondrocytes, or chondrocytes cultured for 48 h. cDNA templates were prepared, and real time RT-PCR was carried out for procollagen *α*1(II) mRNA (b) and Sox9 mRNA (c). (d and e) For collagen II protein synthesis by isolated chondrocytes, cells were washed in Met/Cys-free and serum-free medium for 1 hour. Cells were cultured for a further 6 hours in serum-free medium. Medium was harvested, electrophoresed, and transferred to PVDF for western blotting (d). To detect newly synthesised proteins by autoradiography, cells were washed in Met/Cys-free and serum-free medium for 1 hour, then cultured for 6 hours in Met/Cys-free medium to which [^35^S] Met/Cys was added. Medium was electrophoresed, and gels were silver stained, dried, and exposed to film. The autoradiograph (f) showed 4 bands (A–D) which were cut out and identified by mass spectroscopy. The indicated molecular weights are calculated from the migration of marker proteins. (f and g) Sox9 mRNA stability was measured in juvenile (f) and adult (g) chondrocytes in explants (square) or following isolation (circles). mRNA was measured at indicated times after addition of actinomycin D to the culture medium. Results are representative of 6 adult and 6 juvenile animals. Significance was analysed using Student's *t*-test/ANOVA.

It was possible that the bands detected by western blotting represented preexistent protein leaching from the cartilage explants during culture. To establish whether the differences observed corresponded to newly synthesised collagen, we would have liked to carry out mass spectrometric identification of biosynthetically radiolabelled proteins made during culture. This was however technically impeded by excessive contamination by proteoglycan fragments from the explants electrophoresing in the gel. In order to study collagen protein synthesis, it was necessary to use isolated cells. Confluent chondrocytes were washed in Met/Cys-free culture medium for 1 h. [^35^S] Met/Cys was added and culture continued for 6 h. Medium was harvested, and biosynthetically radiolabelled proteins were electrophoresed, silver stained, and detected by autoradiography (Figure 2(e)). Parallel cultures of the same duration without radiolabel were analysed by western blotting (Figure 2(d)). Four bands which stained with the antibody to collagen II corresponded with four radiolabelled bands (MW 175, 168, 158, and 145 kDa). These radiolabelled bands were excised from dried gels and analysed by mass spectrometry (data not shown). This showed that the slowest band corresponded to the proform of the procollagen *α*1(II) chain because it contained peptides from the N- and C-propeptides as well as from the mature chain. The fastest band contained only peptides of the mature chain. The two intermediate bands were likely to be N-terminally processed and C-terminally processed alpha chains. No biosynthetically labelled bands corresponding to the fully processed procollagen *α*1(II) were detected in the adult sample. Taken together these results showed that the adult cartilage was making less than a tenth of the collagen II protein made by the juvenile tissue.

The other major structural protein of hyaline cartilage besides collagen II is the proteoglycan aggrecan. Aggrecan mRNA levels were similar in adult and juvenile cartilage (Figure 1(c)).

### 3.3. Sox9 Levels Are Greater in Juvenile Cartilage Compared to Adult

Sox9 is a transcription factor that directly regulates collagen II and aggrecan mRNA expression [18]. Sox9 mRNA levels were 3-fold greater and Sox9 protein levels were 2-fold greater in fresh juvenile cartilage compared with adult (Figures 1(e) and 1(f)). Sox5 and Sox6 proteins are cofactors for Sox9 protein; their mRNA and protein levels did not differ significantly between juvenile and adult tissue (data not shown).

### 3.4. Collagen II mRNA Stability

**Figure 3:**
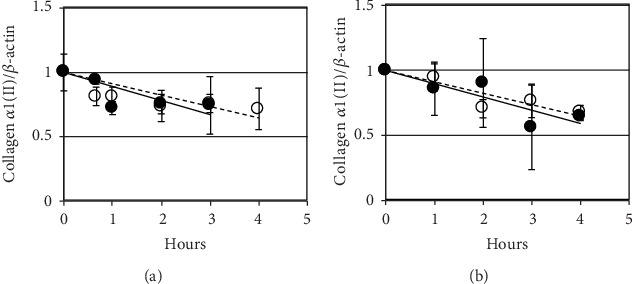
Collagen *α*1(II) mRNA stability in juvenile (solid circles) and adult (clear circles) fresh cartilage and isolated chondrocytes. mRNA stability was measured in freshly dissected explants (a) or following 48 h chondrocyte culture (b) after addition of actinomycin D (10 *μ*g/ml). mRNA was harvested at indicated intervals, cDNA templates were prepared, and real time RT-PCR was carried out for procollagen *α*1(II). Relative gene expression is analysed using ANOVA; lines of best fit are shown. Representative of 8 juvenile and adult pigs.

The difference in procollagen II mRNA levels could be due either to transcription or stability. The stability of procollagen *α*1(II) mRNA (*T*(1/2) ≈ 8 h) was the same in adult and juvenile tissue implying that the differences in steady state levels are largely due to transcription (Figures 3(a) and 3(b)).

### 3.5. Isolation of Adult and Juvenile Chondrocytes from Fresh Cartilage Causes a Fall in Collagen *α*1(II) and Sox9 mRNA Levels

Chondrocytes are most often studied following their isolation from cartilage. The effect of isolation on procollagen *α*1(II) mRNA and Sox9 mRNA expression was studied to see how they changed and whether age-related differences were maintained.

The procollagen *α*1(II) mRNA level was maintained in juvenile cartilage explants cultured for 48 h. The level in the adult explants appeared to increase, but this was variable and not statistically significant (Figure 2(a)). Isolation of chondrocytes did not result in a discernible fall after the 9 h isolation procedure; however, a further 48 h of culture in 10% FCS resulted in a 3-fold drop in procollagen *α*1(II) mRNA in the adult and a 10-fold drop in the juvenile cells. The final difference in mRNA expression between adult and juvenile was 3-fold (Figure 2(b)). Interestingly, Sox9 mRNA levels fell more rapidly since changes were detectable by 9 hours (Figure 2(c)). After 48 hours, Sox9 mRNA had fallen 8-fold in the adult cells and 3-fold in the juvenile.

After 48 h, the adult chondrocytes expressed a third of the amount of collagen II mRNA seen in the juvenile. However, collagen II protein synthesis was easily detected in the cultures of juvenile cells, while no collagen II protein production by the adult cells was detected as judged by either western blotting or biosynthetic labelling (Figures 2(d) and 2(e)). The western blot and autoradiograph bands coincided when overlaid. As described earlier, the fastest band (Figures 2(d) and 2(e), *α*1(II)) was the fully processed *α*1(II) chain, while the slowest band was the pro-*α*1(II) chain because it contained peptides from both N- and C-terminal propeptides. The two intermediate bands (b and c) were presumably partially processed forms of *α*1(II) chains, not prominent in the explant medium samples (Figure 1(d)).

Because of the fall in Sox9 mRNA levels seen upon isolation of the chondrocytes, we investigated a possible change in the half-life of the mRNA. The *T*(1/2) of Sox9 mRNA was ≈4 h in both adult and juvenile cartilage. This fell to 1.5 h in isolated chondrocytes, and there was no difference in this fall between adult and juvenile cells (Figures 2(f) and 2(g)).

## 4. Discussion

Porcine metacarpophalangeal articular cartilage was used because of its availability and consistency of sample age. We found no difference in cell density between the adult and juvenile tissues, but in the adult cartilage there was surface irregularity and the overall thickness was reduced. Liu et al. and Stockwell reported a fall in cell density with increasing age in human and bovine cartilage, respectively, although those studies were made over a greater age range than ours [19, 20]. The thinning of adult cartilage is similar to that previously reported in dogs and pigs [12, 13]. The reduced metachromatic staining suggesting reduced proteoglycan content that we observed in the adult tissue was similar to that reported by Stockwell and Scott in adult human articular cartilage [11].

The reduced collagen II secretion by adult cartilage is likely to be due in part to reduced procollagen *α*1(II) mRNA transcription. Although Sox9 protein is the main transcription factor for procollagen *α*1(II) mRNA, the 2-fold difference in its expression level between the adult and juvenile tissues probably does not explain the age-related difference in collagen II protein synthesis for four reasons. Firstly, there is no age-related difference in the expression of aggrecan mRNA, which is also regulated by Sox9 protein [21]. Secondly, the Sox9 protein level in isolated juvenile chondrocytes is 3-fold less than in fresh cartilage, but the cells are still detectably synthesising collagen II. Thirdly, the difference in procollagen *α*1(II) mRNA levels between juvenile and adult isolated cells is also 3-fold, but the difference in their collagen secretion is much greater than this since no collagen II protein could be detected in the culture medium of adult cells. Fourthly, the isolated juvenile cells and adult cartilage have comparable levels of procollagen *α*1(II) mRNA but only the juvenile chondrocytes can synthesise and secrete collagen II protein. Thus, it appears there is posttranscriptional inhibition of collagen II protein production in the adult cells in addition to reduced transcription. This could be due to inhibition of translation, or the procollagen II mRNA could be translated but the protein degraded either intracellularly or extracellularly. Secreted collagen II protein could be degraded by collagenases (matrix metalloproteinases- (MMP-) 1 and 13) and other nonspecific proteinases [22]. However, we found no significant differences in the mRNA levels of MMP-13 (the main cartilage collagenase), nor any evidence of specific collagenase breakdown products on metabolic labelling in our cultures (data not shown).

The large difference in collagen II protein expression between juvenile and adult is likely to be a significant factor underlying the difference in healing properties of young and old articular cartilage. It is also likely that the relative inability of the adult chondrocytes to make collagen II protein is a factor in the progression of osteoarthritis since the chondrocytes cannot replace lost collagen. In a systematic study from our laboratory of protein synthesis by normal and osteoarthritic cartilage, synthesis of collagen II protein was only detected in osteoarthritic and juvenile samples but never in normal adult cartilage [7]. The level of expression in the osteoarthritic cartilage was, however, very low compared to the juvenile tissue. Thus, collagen II protein synthesis may occur in osteoarthritis but not at levels high enough to restore tissue architecture. These findings are consistent with those of Aigner et al. who reported that procollagen *α*1(II) mRNA levels were increased in osteoarthritic cartilage but Sox9 mRNA levels were reduced [23]. No significant difference was found in Sox5 and Sox6 mRNA and protein levels (cofactors for Sox9 protein).

Many factors apart from the Sox 9 protein level affect collagen II mRNA expression. Various cofactors are required for the activation of the procollagen *α*1(II) promoter by Sox9, including Trap230, PGC-1*α*, LC-Maf, and HIF-2*α* [24–27]. Otero et al. reported that E74-like factor 3 (ELF3) interferes with the collagen II activator function of CBP/300 and Sox9, and they found increased expression of ELF3 in osteoarthritic chondrocytes correlated with methylation of its promoter site [28]. Sox9 protein and Sirtuin 1, a histone deacetylase, activate the enhancer/promoter sites of collagen *α*1(II) increasing collagen expression [29]. Sox9 protein also regulates the BBF2H7 transcription factor that enhances collagen II protein transport from the endoplasmic reticulum to the Golgi apparatus regulating its secretion [30]. From an aging perspective, epigenetic mechanisms such as histone modification, DNA methylation, and, noncoding RNAs may also be important [31, 32].

In addition to the age-related differences in chondrocyte function, we also studied the effect of isolating cells from cartilage. We found that Sox9 mRNA levels fell within 9 hours of cell isolation, preceding the fall in collagen II mRNA levels. The fall in Sox9 mRNA may be due to its destabilisation. The half-life in isolated porcine chondrocytes is similar to that of 1.8 h reported by Tew and Clegg in isolated human chondrocytes [33]. Interestingly, Tew and Hardingham [34] reported that inhibiting p38 MAPK using SB202190 destabilised Sox9 mRNA suggesting that p38 MAPK may stabilise the Sox9 mRNA. The fall in Sox9 mRNA upon isolation of chondrocytes may partly explain the fall in collagen II mRNA. The role of MAPKs in Sox9 and collagen II protein expression requires further investigation.

Ono et al. studied human articular chondrocytes taken from the osteoarthritic knee joint of patients aged 56-86 years [10]. They compared mRNA expression in cartilage slices with that in isolated chondrocytes that had reached confluence following plating at 1 × 10^4^ cells/cm^2^. No drop in collagen II, Sox9, and aggrecan mRNA was observed. Possibly, the osteoarthritic cartilage cells were already dedifferentiated prior to isolation.

Taken together our results indicate that the very marked decrease in collagen II protein secretion that occurs on maturation to the adult is due to both transcriptional and posttranscriptional regulation. The reduction in transcription of procollagen *α*1(II) mRNA may in part be due to a reduction in Sox9 protein level. This is a modest reduction, perhaps 2-fold, and does not affect the expression of aggrecan, another cartilage-specific molecule. Thus, other transcriptional regulators are probably involved. The mechanism of the posttranscriptional regulation remains to be ascertained, but translational regulation is likely.

We have also shown that collagen II expression falls sharply when chondrocytes are removed from their matrix. This was associated with a destabilisation of Sox9 mRNA, which preceded the fall in procollagen *α*1(II) mRNA levels. Thus, posttranscriptional control of Sox9 likely underlies the fall in collagen expression. It is conventional to consider the collagen expression of confluent primary cultures (P0) as being maximal or 100%, and the decline in collagen II expression upon serial passaging of the cells has been much studied. Our results suggest the collagen expression in P0 culture is much reduced when compared to that found in intact tissue.

An understanding of why collagen II protein expression is silenced in the adult cartilage, with focus on the transcriptional and posttranscriptional mechanisms could lead to improved cartilage repair and regenerative techniques.

## Data Availability

Data is available from the host institution for review if required.
